# Progressive disseminated histoplasmosis mimicking a flare of systemic lupus erythematosus: a European case report

**DOI:** 10.1099/jmmcr.0.005035

**Published:** 2016-08-30

**Authors:** M. L. M. van Doorn-Schepens, E. J. Peters, R. M. van Vugt, J. I. van der Spoel, K. van Dijk

**Affiliations:** ^1^​ Department of Medical Microbiology and Infection Control, VU University Medical Center, Amsterdam, the Netherlands; ^2^​ Department of Internal Medicine, VU University Medical Center, Amsterdam, the Netherlands; ^3^​ Department of Rheumatology, VU University Medical Center, Amsterdam, the Netherlands; ^4^​ Intensive Care Unit, VU University Medical Center, Amsterdam, the Netherlands

**Keywords:** histoplasmosis, disseminated, amphotericin B

## Abstract

**Introduction:**

Diagnosing progressive disseminated histoplasmosis (PDH) in patients with systemic lupus erythematosus (SLE) is diagnostically challenging. Since PDH is lethal when untreated, awareness of this infection in patients with SLE is of utmost importance. To the best of our knowledge, this is the first description of a case of PDH in a patient with SLE in Europe.

**Case presentation:**

A 56-year-old woman of Surinamese descent with a history of SLE, presented with fever and polyarthritis. Although a flare of SLE was suspected initially, cultures of bone marrow and broncho-alveolar lavage fluid grew *Histoplasma capsulatum*.

**Conclusion:**

This case report highlights that physicians should be aware of progressive disseminated histoplasmosis in patients with SLE treated with immunosuppressive agents. The signs and symptoms can easily mimic a SLE flare, which would then be treated with more aggressive immunosuppression. Failure to recognize the infection will therefore invariably lead to death of the patient. Progressive disseminated histoplasmosis is usually not recognized by doctors in non-endemic areas such as Europe. However, globalisation and more frequent intercontinental traffic of immunocompromised patients currently increases the incidence of histoplasmosis in these areas. It is therefore of life-saving importance that doctors are aware of the features of the infection in areas where *H. capsulatum* is not endemic.

## Introduction

*Histoplasma capsulatum* is a soil-based fungus which is mostly associated with river valleys (e.g. Ohio and Mississippi river valleys), and places where bats live and birds roost, predominantly in (sub) tropical regions. After inhalation of spores, this microorganism can cause a broad range of clinical manifestations. The vast majority of infections (>90 %) are asymptomatic, but symptoms ranging from an influenza-like illness to death are also described (Goodwin *et al*., 1981). Contributing factors to the extent of the disease are higher inoculum size, increasing age and underlying disease. The use of immunosuppressive drugs is a risk factor for disseminated or fatal disease (Wheat *et al*., 1982). Adequate cell-mediated immunity is necessary to control a *Histoplasma* *capsulatum* infection after exposure (Porta & Maresca, 2000). Patients with systemic lupus erythematosus (SLE) have defects in their humoral and cellular immune systems (Tsokos, 1994). The combination of intrinsic immune system defects with chronic immunosuppressive therapy makes patients with SLE prone to develop progressive disseminated histoplasmosis (PDH) when infected. Although the reported incidence of invasive fungal infections in patients with SLE is low, 0.64–1.04 % (Weng *et al*., 2010), timely recognition of PDH is important because of the high mortality if untreated. Diagnosis is frequently delayed because symptoms are often attributed to SLE (Lim *et al*., 2013). Here we describe the first reported case of disseminated histoplasmosis in a patient with SLE in Europe.

## Case report

**Fig. 1.**
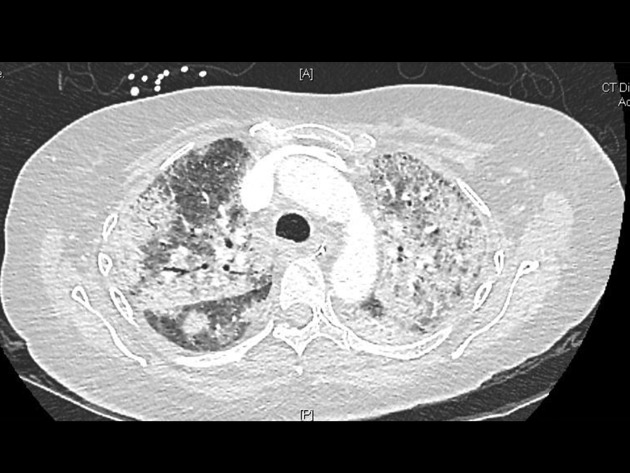
Thoracic computed tomography (CT) of the lungs showing diffuse ground glass opacities and bilateral consolidations in the upper pulmonary lobe.

**Fig. 2.**
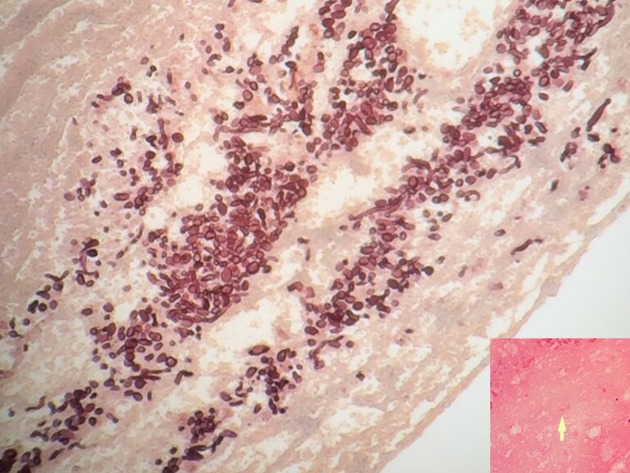
Histopathology of the gingival biopsy showing yeast buds and septate hyphae (haematoxylin-eosin stain). The inlet is a periodic acid-Schiff stain of the gingival biopsy showing encapsulated yeast cells.

A 56-year-old woman of Surinamese descent with a history of SLE and splenectomy was admitted to the Department of Rheumatology at the VU University Medical Center in Amsterdam because she had collapsed and had a fever. Her last visit to Suriname was in 2008. She had been treated with mycophenolate mofetil 1250 mg orally (PO) twice daily and prednisone 20 mg PO once daily. The dosage of both these drugs was recently increased because of exacerbation of her SLE-nephritis with a nephrotic syndrome. On admission, her temperature was 39.1 °C. She had polyarthritis with swelling and calor of her left knee and interphalangeal joints of her right hand. The white blood cell count was 7.9 × 10^9^ l^−1^ (segmented neutrophils 7.50 × 10^9^ l^−1^, lymphocytes 0.24 × 10^9^  l^−1^), haemoglobin 7.5 mmol l^−1^, platelet count 419 × 10^9^ l^−1^, and C-reactive protein 210 mg l^−1^. Nitrite in urine was positive. Antibiotic treatment was started with intravenous ceftriaxone, 2 g once a day, because a complicated urinary tract infection was suspected. Within one week of admission, the patient was admitted to the ICU because of respiratory insufficiency, and mechanical ventilation was started. Computed tomography (CT) of the lungs showed segmental pulmonary embolism, diffuse ground glass opacities and bilateral consolidations in the upper pulmonary lobes (Fig. 1). She also had progressive liver failure; bilirubin was 35 µmol l^−1^, alkaline phosphatase 950 U l^−1^, gamma-glutamyl transferase 6335 U l^−1^, aspartate aminotransferase 116 U l^−1^, and alanine transaminase 273 U l^−1^. A CT-scan of the abdomen revealed no explanation for the liver failure. The culture of the broncho-alveolar lavage (BAL) fluid was negativein the first days. PCRs for *Legionella pneumophila, Mycobacterium tuberculosis*,* M. genus*,* Chlamydia pneumoniae*,* Chlamydia psittaci*,* P*neumocystis* jiroveci*, cytomegalovirus, Epstein-Barr virus and respiratory viruses were negative. With these negative microbiological test results, clinical deterioration of the patient was attributed to an exacerbation of SLE, and subsequently intravenous methylprednisolone pulse therapy was started in a daily dose of 1000 mg for three days. At first, her clinical condition improved and she was extubated. However within three days she was increasingly somnolent, in respiratory failure and she was intubated again. CT-scans of the lungs showed profound progression of the ground glass opacities. Histoplasmosis was considered in the differential diagnosis. However, at that moment fungal culture was negative, and with a low likelihood of *H. capsulatum*, no (toxic) pre-emptive therapy was initiated. A course of methylprednisolone pulse therapy was started again, but to no avail. Nine days after performing the BAL, culture of the BAL fluid grew a fungus, which was determined as *H. capsulatum* by the dimorphic character of the fungus, with a mycelial phase at 37 °C and a yeast phase at 30 °C. PCR performed on a bone marrow biopsy and culture of this biopsy were both positive for *H. capsulatum*. Antifungal therapy with liposomal amphotericin B (3.0 mg kg^−1^ daily) was started to treat this PDH, and immunosuppressive therapy was reduced to 20 mg prednisone per day. Two weeks after starting antifungal therapy the patient developed a lesion resembling pyogenic granuloma in her oral cavity from which she bled profoundly. A periodic acid-Schiff stain (PAS stain) of a needle biopsy of the gingiva showed encapsulated yeast cells and a haematoxylin-eosin stain of this biopsy showed 2–4 µm yeast buds and septated hyphae (Fig. 2). The patient could slowly be weaned from mechanical ventilation, and after one month on the ICU, she was extubated. After four weeks, liposomal amphotericin B was switched to itraconazole PO, 200 mg three times daily for three months, and then 200 mg once daily as lifelong suppressive therapy. Three months after she was diagnosed with PDH and treatment was started, the patient could be discharged from the hospital. Two months after her discharge her liver and kidney function tests had fully recovered.

## Discussion

Symptoms of disseminated histoplasmosis mimic SLE-related symptoms, with focal lesions in the same organs affected by SLE. As a consequence, disseminated histoplasmosis is a diagnostic challenge for physicians. A complicating factor is that culturing *H. capsulatum* can take seven days to three weeks. *H. capsulatum* antigen testing on urine or serum can lead to a faster diagnosis with a reported sensitivity of 95–100 % (Connolly *et al*., 2007; Hage *et al*., 2010, 2011). As a consequence, diagnosing PDH solely by fungal culture can lead to a significant delay in initiating adequate antifungal therapy. In case the treating physician does not consider disseminated histoplasmosis, the dose of corticosteroids will likely be increased to treat a suspected flare of SLE. When symptoms temporarily improve by the increase in dosage of steroids, clinicians are put on the wrong track even more; the response to steroids affirms the initial diagnosis of SLE exacerbation. Even though the diagnosis of PDH was already considered in this patient three weeks after admission, a slight increase of SLE activity could not be excluded and symptoms were initially attributed to SLE. A case-based review on PDH in patients with SLE suggested that there is significant delay from onset of symptoms to time of diagnosis with a median time of 6.5 months (range 0.75–31 months) (Lim *et al*., 2013). Most of the patients in that review were using chronic immunosuppression, most commonly steroids with 56.3 % of the patients taking a dose of 20 mg prednisone or higher per day. In four out of the five fatal cases, the patients were using additional immunosuppressant drugs, like mycophenolate mofetil. Since the spleen plays an important role in our immune response it would be plausible that (functional) asplenia makes a patient prone to infection with *H. capsulatum*. However, splenectomy or asplenia does not seem to be a risk factor for an infection with *H. capsulatum*. Only sporadic cases have been reported of *H. capsulatum* infection in asplenic patients (Naina *et al*., 2010; Rose *et al*., 1982; Stone *et al*., 1990). Most case reports of disseminated histoplasmosis in patients with SLE are described in high-endemic areas (Table 1) (Lim *et al*., 2013). The patient in this case report is of Surinam descent, where *H. capsulatum* is also high-endemic (Colombo *et al*., 2011). In such high-endemic areas, physicians are more aware of PDH in patients with SLE than physicians in non-endemic areas. This case report describes the first reported case of disseminated histoplasmosis in a patient with SLE in Europe. The difficult diagnosis of PDH and the low awareness of European clinicians might well have resulted in under-reporting of PDH in European medical literature. Furthermore, the diagnosis was (partially) based on a positive fungal culture of BAL-fluid. The classical histopathological findings of the gingival biopsy showing yeast buds and septate hyphae and encapsulated yeast cells is a rarity. These findings make it a unique case.

**Table 1.** Cases of progressive disseminated histoplasmosis in systemic lupus erythematosus patients Clinical data of 17 cases described in the literature, plus the data of the current case report. PDH, Progressive disseminated histoplasmosis; SLE, systemic lupus erythematosus; M, male; F, female; u/a, unavailable; D, death; S, survived; bx, biopsy; hpe, histopathologic examination; cx, culture.

Age (years)/ gender	Symptom	SLE flare	Antifungal treatment	Outcome	Initial presentation to diagnosis (time)	Initial method of diagnosis	Dose of steroid per day at onset of symptoms	Other immunosuppressants at onset of symptoms
49/F	Dyspnea, productive cough, pleurisy, night sweats, weight loss	Yes	Amphotericin B	D	4–5 months	Autopsy	Prednisone >20 mg	
22/F	Fever, vision change	u/a	Amphotericin B	D	u/a	Autopsy	Steroids, dose not specified	Azathioprine
23/F	Fever, night sweats, lethargy, pleurisy	No	Amphotericin B	S	7 months	Histoplasmosis fixation antibody titer 1:128	Prednisone 40–60 mg	
65/M	Fever, fatigue, dyspnea	u/a	Amphotericin B	D	u/a	Bone marrow bx	Prednisone 20–60 mg	6-Mercaptopurine
23/F	Fever, malaise, weight loss	u/a	Amphotericin B	S	u/a	Liver bx	Prednisone 20–60 mg	
35/M	Fever, malaise, dyspnea	u/a	No treatment	D	u/a	Autopsy	Prednisone 20–60 mg	6-Mercaptopurine
56/F	Fever, rash, headache, myalgia	Yes	Amphotericin B, fluorocytosin	S	3 weeks	hpe (skin bx)	No steroids	
53/F	Fever, confusion, fatigue, weakness, jaundice, papulonodular skin lesion	Yes	Amphotericin B, itraconazole	S	6–7 months	cx (bone marrow bx)	Prednisone 30–80 mg	
39/F	Headache, confusion	No	Amphotericin B, itraconazole	S	6.5 months	hpe (cerebrospinal fluid)	Prednisone 20 mg	
32/F	Fever, night sweats, lethargy, weight loss, irregular menses (ovarian dysfunction)	No	Ketoconazole	S	2 years 7 months	hpe (ovary-surgical specimen)	Prednisone 10 mg	
49/F	Nasal ulceration, cutaneous ulcer	u/a	Amphotericin B, itraconazole, voriconazole	S	2 years	hpe (cutaneous ulcer, nasal ulceration bx)	Prednisone 5 mg	
47/F	Nasal ulceration	No	Ketoconazole	S	u/a	cx (nasal septum bx)	Steroids, dose not specified	
35/F	Hoarseness	u/a	Amphotericin B, itraconazole	S	7 months	cx (nasal crusts)	Prednisone 10 mg	
48/F	Fever, malaise, fatigue and arthralgias, swelling and stiffness of left hand, swelling of left ankle	Yes	Amphotericin B, miconazole	D	6 months	hpe (palmar mass surgical specimen)	Methylprednisolone 16 mg	Azathioprine
42/F	Swelling and stiffness of index finger of left hand	No	Itraconazole	S	3 months	cx (synovium bx)	No steroids	
32/F	Swelling, erythema in right forearm	No	Itraconazole	S	2 months	cx (synovium bx)	Prednisone 30 mg	Mycophenolate mofetil
56/F	Fever and polyarthritis	Yes	Liposomal Amphotericin B, itraconazole	S	3 weeks	PCR (bone marrow bx), cx (BAL), hpe (gingival bx)	Prednisone 20 mg	Mycophenolate mofetil

In conclusion, this case report highlights that physicians should be aware of progressive disseminated histoplasmosis in patients with SLE, treated with immunosuppressive agents. The signs and symptoms can easily mimic a SLE flare, which would then be treated with more aggressive immunosuppression. Failure to recognize the infection will therefore invariably lead to death of the patient. Progressive disseminated histoplasmosis is usually not recognized by doctors in non-endemic areas such as Europe. However, globalisation and more frequent intercontinental traffic of immunocompromised patients currently increases the incidence in these areas. It is therefore of life-saving importance that doctors in areas where *H. capsulatum* is not endemic are aware of the features of the infection, especially in patients from endemic areas. By creating awareness of this infection, faster diagnostic tests can be used to diagnose PDH. In this particular case, specific fungal staining, such as methenamine silver or periodic acid-Schiff stains (PAS), could have been performed on the BAL fluid or a bone marrow aspirate. Furthermore *H. capsulatum* antigen testing could have been performed on urine or serum for faster diagnosis (Connolly *et al*., 2007; Hage *et al*., 2010, 2011).
